# Postnatal development of cerebellar zones revealed by neurofilament heavy chain protein expression

**DOI:** 10.3389/fnana.2013.00009

**Published:** 2013-05-09

**Authors:** Joshua J. White, Roy V. Sillitoe

**Affiliations:** ^1^Department of Pathology and Immunology, Baylor College of Medicine, Jan and Dan Duncan Neurological Research Institute of Texas Children's HospitalHouston, TX, USA; ^2^Department of Neuroscience, Baylor College of Medicine, Jan and Dan Duncan Neurological Research Institute of Texas Children's HospitalHouston, TX, USA

**Keywords:** purkinje cells, patterning, topography, circuit, development

## Abstract

The cerebellum is organized into parasagittal zones that control sensory-motor behavior. Although the architecture of adult zones is well understood, very little is known about how zones emerge during development. Understanding the process of zone formation is an essential step toward unraveling how circuits are constructed to support specific behaviors. Therefore, we focused this study on postnatal development to determine the spatial and temporal changes that establish zonal patterns during circuit formation. We used a combination of wholemount and tissue section immunohistochemistry in mice to show that the cytoskeletal protein neurofilament heavy chain (NFH) is a robust marker for postnatal cerebellar zonal patterning. The patterned expression of NFH is initiated shortly after birth, and compared to the domains of several known zonal markers such as zebrin II, HSP25, neurogranin, and phospholipase Cβ4 (PLCβ4), NFH does not exhibit transient expression patterns that are typically remodeled between stages, and the adult zones do not emerge after a period of uniform expression in all lobules. Instead, we found that throughout postnatal development NFH gradually reveals distinct zones in each cerebellar lobule. The boundaries of individual NFH zones sharpen over time, as zones are refined during the second and third weeks after birth. Double labeling with neurogranin and PLCβ4 further revealed that although the postnatal expression of NFH is spatially and temporally unique, its pattern of zones respects a fundamental and well-known molecular topography in the cerebellum. The dynamics of NFH expression support the hypothesis that adult circuits are derived from an embryonic map that is refined into zones during the first 3-weeks of life.

## Introduction

The adult cerebellum is comprised of relatively few cell types that are found, with only few exceptions, throughout all ten of its lobules (Larsell, 1952; Altman and Bayer, 1997; Mugnaini et al., 2011). However, underlying its apparently uniform cellular architecture is an elaborate array of sagittal zones that divide the entire cerebellum into an exquisitely organized topographic map (Apps and Hawkes, 2009). For example, zebrin II/AldolaseC, the best known molecular marker of cerebellar zones, reveals an alternating pattern of Purkinje cells in the adult cerebellum (Brochu et al., 1990; Ahn et al., 1994; Sillitoe and Hawkes, 2002; White and Sillitoe, 2012). Interestingly, compared to zebrin II, other markers such as the small 25 kDa heat shock protein, HSP25, reveal a distinct pattern of zones in different lobules (Armstrong et al., 2000). Because zonal patterns are unique to subsets of lobules, molecular patterning may be used to further partition the cerebellum into four transverse domains: anterior (AZ: ~lobules I–V), central (CZ: ~lobules VI–VII), posterior (PZ: ~lobules VIII–dorsal IX), and nodular (NZ: ~lobules IX ventral and X) (Ozol et al., 1999).

Recent work suggests that adult zones are likely derived from a simpler pattern of embryonic “clusters” (Ozol et al., 1999; Larouche et al., 2006; Sillitoe et al., 2009; Namba et al., 2011; Fujita et al., 2012). However, we still do not fully understand how developmental clusters transform into mature zones with well-defined boundaries. From approximately embryonic day (E) 14, Purkinje cell clusters begin to express a variety of molecular markers that compartmentalize the developing cerebellum into an array of nascent sagittal zones (Wassef and Sotelo, 1984; Wassef et al., 1985; Oberdick et al., 1993; Millen et al., 1995; Nunzi et al., 1999; Ozol et al., 1999; Armstrong et al., 2001; Larouche et al., 2006; Furutama et al., 2010; Redies et al., 2011). Although we now know that the adult pattern reflects a complicated correlate of the embryonic cluster map, and apparently is derived from approximately 50 clusters (Fujita et al., 2012), it is still not clear how the early postnatal plan transforms into the mature zonal map. Several roadblocks have hampered progress in understanding zone formation during the first three weeks after birth. First, most developmental patterns only transiently reveal a specific set of zones; embryonic cluster markers are either suppressed or eventually mark all Purkinje cells (calbindin, *engrailed1/2*, *L7/Pcp2*, White and Sillitoe, 2012). Second, early postnatal “bridge” markers such neurogranin (Larouche et al., 2006) and phospholipase Cβ4 (PLCβ4; Marzban et al., 2007; Young and Kothary, 2011) do not reveal zones in the vermis and hemispheres throughout postnatal development, making it hard to examine how each stage of development is ultimately linked to the adult pattern of zones. Third, the onset of adult stripe patterns occurs only after postnatal day (P) 15 (Armstrong et al., 2001; Apps and Hawkes, 2009), which typically leaves a gap between when cluster and bridge makers delineate zones and when the well-understood framework of zebrin-like patterns are observed. In this study, we fill this gap in our knowledge by providing insight into how the postnatal zonal map emerges using the expression of neurofilament heavy chain (NFH), a novel marker for zonal patterns in the cerebellum.

The neurofilaments are a sub-family of proteins that comprise part of a larger family of intermediate cytoskeletal proteins (Young and Kothary, 2011). The three members are named according to their sizes: neurofilament light (NFL, 68 kDa), neurofilament medium (NFM, 160 kDa), and NFH (205 kDa) (Perrot et al., 2008). The neurofilament proteins are differentially expressed during development. NFL and NFM are expressed during the early stages of synaptogenesis and axon targeting, while NFH is predominantly expressed in the postnatal brain and specifically during circuit stabilization (Carden and Trojanowski, 1987), where it may function as a molecular indicator of cytoskeletal and cell maturation (Grant and Pant, 2000; Lariviere and Julien, 2004). In the cerebellum, NFH is expressed in Purkinje cells (Demilly et al., 2011), neurons of the cerebellar nuclei (Jankovski et al., 1996; Hoshino et al., 2005; Demilly et al., 2011), and the axons of basket cell interneurons (Demilly et al., 2011).

We recently showed that the expression of non-phosphorylated NFH divides all lobules of the adult mouse cerebellum into a complex map of parasagittal Purkinje cell zones (Demilly et al., 2011). Because the onset of NFH expression coincides with critical stages of circuit formation, and because its expression is modulated by sensory input (Duffy et al., 2007), we postulated that NFH expression might also be associated with postnatal Purkinje cell zone formation, a key step required for the construction of cerebellar sensory-motor circuitry. We show that NFH expression in Purkinje cells is initiated perinatally in a sagittal pattern that resolves into clear zones by the end of the first postnatal week. During the second and third postnatal weeks, NFH expression continues to mark the same set of zones, albeit with sharper resolution of each zonal boundary. Remarkably, at all stages examined NFH expression clearly delineates zones in all lobules of the vermis and in the hemispheres. These data suggest that despite the dynamic morphogenetic patterning that transforms embryonic clusters to adult zones (Larouche et al., 2006; Fujita et al., 2012), a stable map of sagittal compartments may link cerebellar development to adult function. Such a map might be essential for guiding circuit connectivity and perhaps provide a fundamental scaffold upon which synaptic pruning and plasticity shape sensory-motor circuits.

## Methods

### Animals

All animal studies were carried out under an approved IACUC animal protocol according to the institutional guidelines at Baylor College of Medicine. Male and female outbred Swiss Webster (Taconic, Albany, NY, USA) mice were maintained in our colony and used for all experiments. Pups were collected at P0, P1, P2, P3, P5, P7, P10, P12, P15, P17, and P20. For these studies, mice were considered adult after P28 because by this age cerebellar patterns are mature. Noon on the day a vaginal plug was detected was considered embryonic day (E) 0.5. At least three mice were used for each age.

### Immunohistochemistry

**Figure 1:**
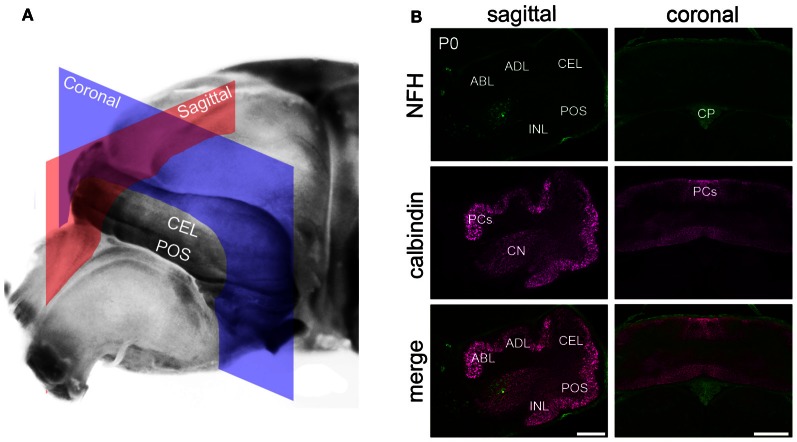
**NFH is expressed in the cerebellar nuclei of newborn mice. (A)** An unstained brain at P0 illustrating the cutting planes used to acquire the tissue sections shown in panel **(B)**. The sagittal plane is shown in red and the coronal plane in blue. **(B)** NFH labels the CN and not Purkinje cells. Purkinje cells were identified with Calbindin, a specific marker for Purkinje cells. Background staining was detected in the choroid plexus. Abbreviations: CN, cerebellar nuclei; CP, choroid plexus; NFH, neurofilament heavy chain; P0, postnatal day 0; PCs, Purkinje cells; and the cardinal lobes are ABL, anterobasal lobe; ADL, anterodorsal lobe; CEL, central lobe; POS, posterior lobe; INL, inferior lobe. The cardinal lobes were named according to the nomenclature of Altman and Bayer (1997). Scale bar = 200 μm for the sagittal sections and = 500 μm for the coronal sections.

Mice were anesthetized with avertin and perfused with 4% paraformaldehyde (PFA) diluted in 0.1 M phosphate-buffered saline (PBS; pH 7.2). The tissue was then post-fixed for 24–48 h in 4% PFA and then cryoprotected in a series of sucrose solutions (15% and 30%, both diluted in PBS). Serial 40 μm thick coronal and sagittal sections (Figure 1A) were cut on a cryostat and collected as free-floating sections in PBS. Immunohistochemistry was carried out as described previously (Sillitoe et al., 2003, 2008). Briefly, tissue sections were washed thoroughly, blocked with 10% normal goat serum (NGS; Sigma, St. Louis MO, USA) for 2 h at room temperature (RT) and then incubated in 0.1 M PBS containing 10% NGS, 0.1% Tween-20 and the primary antibodies (see below) for 16–18 h at RT. The tissue sections were then washed three times in PBS and incubated in secondary antibodies for 2 h at RT. The tissue was rinsed again and immunoreactivity revealed as listed below.

### Wholemount immunohistochemistry

Wholemount immunohistochemistry for NFH was carried out as previously described (Sillitoe and Hawkes, 2002), with a few alterations (White et al., 2012). Cerebella were first post-fixed in Dent's fixative for 5–6 h at RT and then incubated in Dent's bleach at 4°C. They were then dehydrated twice in 100% MeOH for 30 min each and subjected to four cycles of freezing in 100% MeOH and thawing to RT, and then incubated overnight in 100% MeOH at −80°C. The following day the tissue was rehydrated for 90 min each in 50% and 15% MeOH in PBS at RT. For cerebella from mice older than P7, we enzymatically digest the cerebellum in 10 ug/ml proteinase K for 2–3 min at RT. Cerebella were then rinsed three times for ten minutes in PBS at RT and blocked by incubating in PBSMT for 6–8 h. Primary antibodies were diluted in PBSMT containing 5% DMSO and incubated for 48 h at 4°C. After this incubation, wholemounts were washed in PBSMT three times for 2–3 h each and then incubated overnight in secondary antibodies diluted in PBSMT and 5% DMSO at 4°C. After secondary antibodies the tissue was washed three times in PBSMT for 2–3 h each and then incubated in PBSMT overnight to ensure removal of unbound antibodies. Cerebella were rinsed in PBT for 2 h at RT and then subjected to the diaminobenzidine (DAB; see below) reaction until an optimal level of staining intensity was achieved.

### Antibodies

Mouse monoclonal anti-NFH (also called anti-SMI-32; 1:1500) was purchased from Covance (Princeton, NJ). Anti-SMI-32 recognizes the non-phosphorylated form of NFH (see manufacturer product datasheet for details), which on tissue sections labels the soma, dendrites, and axons of adult Purkinje cells (Demilly et al., 2011). Rabbit polyclonal anti-PLCβ4 (1:250) recognizes a subset of Purkinje cells and unipolar brush cell interneurons (Sarna et al., 2006; Marzban et al., 2007; Chung et al., 2009) and was purchased from Santa Cruz Biotechnology (Santa Cruz, CA: catalog #sc-20760). Rabbit anti-neurogranin (1:500) was raised against full-length recombinant rat neurogranin protein (Chemicon, Temecula, CA: catalog #AB5620). Neurogranin recognizes Purkinje cells in the neonatal cerebellum and Golgi cells in the adult cerebellum (Singec et al., 2003; Larouche et al., 2006). Rabbit anti-calbindin (1:20,000) is heavily expressed in Purkinje cells (Celio, 1990) and was produced against recombinant rat calbindin D28-K (Swant, Bellinzona, Switzerland). In the current study, each antibody revealed a staining pattern that was identical to what has previously been described.

We visualized the localization of immunoreactive complexes using DAB (0.5 mg/ml; Sigma, St. Louis, MO, USA) as a chromogen. These experiments were achieved using horseradish peroxidase (HRP) conjugated goat anti-rabbit or HRP-conjugated goat anti-mouse (both diluted 1:200 in PBS; DAKO, Carpinteria, CA, USA) secondary antibodies. Staining for fluorescence immunohistochemistry was carried out using Alexa 488- and 555-conjugated immunoglobulins (Molecular Probes Inc., Eugene, OR, USA), both diluted 1:1,500.

### Microscopy

Photomicrographs of tissue sections were captured using a Leica DFC360 FX (fluorescence) and DFC 490 (DAB reacted tissue sections) camera mounted on a Leica DM6000 microscope. Images of tissue sections were acquired and analyzed using Leica Application Suite and Leica Application Suite FX software. Photomicrographs of wholemount stained cerebella were captured with a Leica MZ16 FA stereomicroscope mounted with a Leica DFC3000 FX camera running Leica LAS software supplemented with the Leica Montage module. All raw data was imported into Adobe Photoshop CS4 and corrected for brightness and contrast levels only. Schematics were drawn in Adobe Illustrator CS4.

## Results

In this study, we performed wholemount and tissue section immunohistochemistry to determine the spatial and temporal expression of NFH patterning during postnatal development of the mouse cerebellum. Two forms of NFH are expressed in the cerebellum: phosphorylated and non-phosphorylated (Marc and Clavel, 1986; Langley and Sternberger, 1988; Vega et al., 1994). Because the phosphorylated form is primarily expressed in axons, we used the non-phosphorylated form (labeled with SMI-32) to investigate cerebellar architecture because it is heavily expressed in somata, dendrites, and axons (Marc and Clavel, 1986), and because it marks Purkinje cell zones in the adult cerebellum (Sillitoe et al., 2008; Demilly et al., 2011).

### NFH expression reveals a consistent array of zones throughout postnatal development

We examined the cerebellum at several key stages during postnatal development (see Methods) to gain a better understanding of how zones resolve during circuit formation. Notably, we discuss in detail our findings from P0 to P2 mice because several zonal markers including zebrin II, HSP25, and PLCβ4, initiate expression around birth. We examine P7 in detail not only because it marks the end of the first week, but also because it correlates with when the major mossy fiber tracts are resolving into zones (Arsenio Nunes and Sotelo, 1985; Sillitoe et al., 2010; Reeber et al., 2012), and because one of the most powerful connections in the brain, the climbing fiber-Purkinje cell synapse, begins to be pruned to its mature configuration (Hashimoto and Kano, 2005; Bosman et al., 2008; Kano and Hashimoto, 2009; White and Sillitoe, 2012). We discuss P12 because at this age markers such as zebrin II and HSP25 exhibit a transient stage of uniform expression in all regions of the cerebellum (Armstrong and Hawkes, 2000). We analyze P20, the end of postnatal week three, because by this age the adult zonal patterns are in place, afferent pruning is essentially complete, and the maturation of cerebellar lobule morphology is near completion (Sillitoe and Joyner, 2007).

#### Postnatal week 1

**Figure 2:**
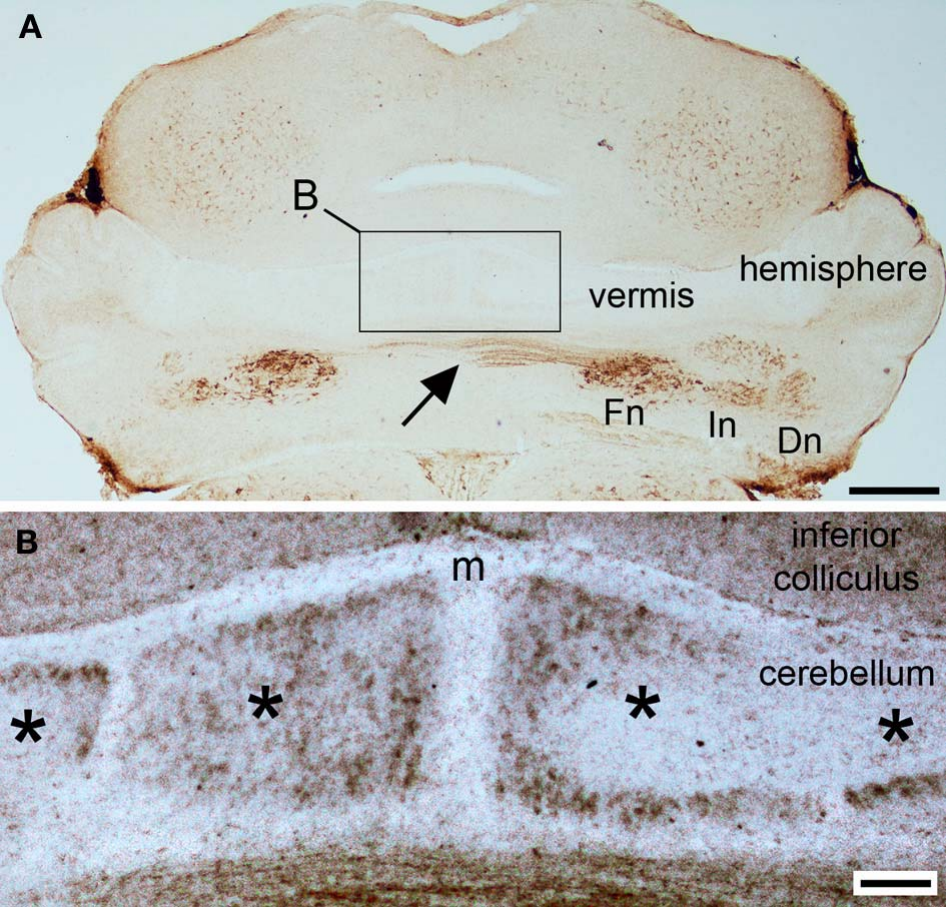
**NFH is expressed in parasagittal zones at postnatal day 2. (A)** At P2, NFH is heavily expressed in all three sets of cerebellar nuclei [fastigial nucleus (Fn), interpositus nucleus (In), dentate nucleus (Dn)], in the commissural axons from the Fn (arrow), and weakly in broad zones of Purkinje cells in the vermis and hemispheres. **(B)** High power image of the midline (m) showing Purkinje cell zones (asterisks). Scale bar in **(A)** = 500 μm and **(B)** = 100 μm.

In general, NFH expression increases gradually as the brain develops (Schlaepfer and Bruce, 1990). In the cerebellum, at postnatal day (P) 0/1 (Figure 1A) we observed selective expression of NFH only in neurons of the cerebellar nuclei (Hoshino et al., 2005; Figure 1B). As in the adult cerebellum, NFH expression delineates the architecture of all three divisions of the deep nuclei; fastigial, interposed, and dentate (Demilly et al., 2011; Figures 1 and 2A). In each division of the cerebellar nuclei, NFH immunoreactivity has been shown to mark large glutamatergic neurons (Hoshino et al., 2005; Fink et al., 2006).

Purkinje cell expression of NFH is thought to increase during early postnatal development (Marc and Clavel, 1986; Riederer et al., 1996). Consistent with these previous data, we did not detect NFH expression in Purkinje cells at P0 or at P1 (Figure 1B). However, Purkinje cell expression of NFH is observed from P2 onwards (Figure 2). Importantly, at P2/3 Purkinje cell expression of NFH initiates in a series of zones. Notably however, Purkinje cell expression of NFH is weak (although immunoreactivity can be seen at high power in Figure 2B), the lateral margins of each zone are not sharply delineated, and zonal boundaries are poorly resolved (Purkinje cell clusters are marked by asterisks in Figure 2B).

**Figure 3:**
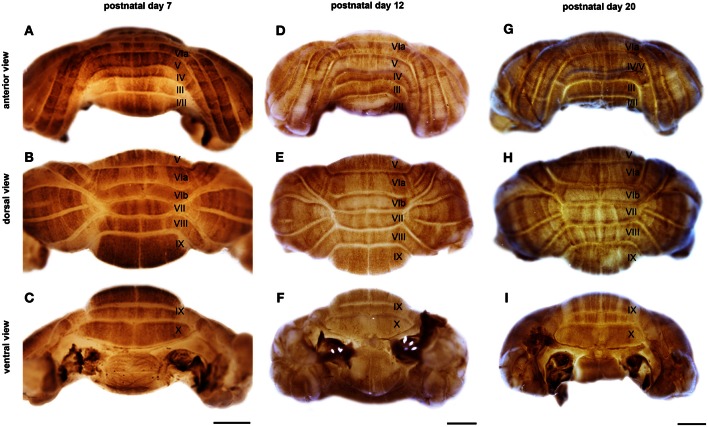
**NFH is expressed in zones throughout postnatal development.** Wholemount immunohistochemistry demonstrates that NFH is expressed in zones of Purkinje cells at P7 **(A–C)**, P12 **(D–F)**, and P20 **(G–I)**. Note that all regions of the vermis and hemispheres are compartmentalized into zones. The vermis lobules are numbered with Roman numerals. Scale bars = 1 mm.

**Figure 4:**
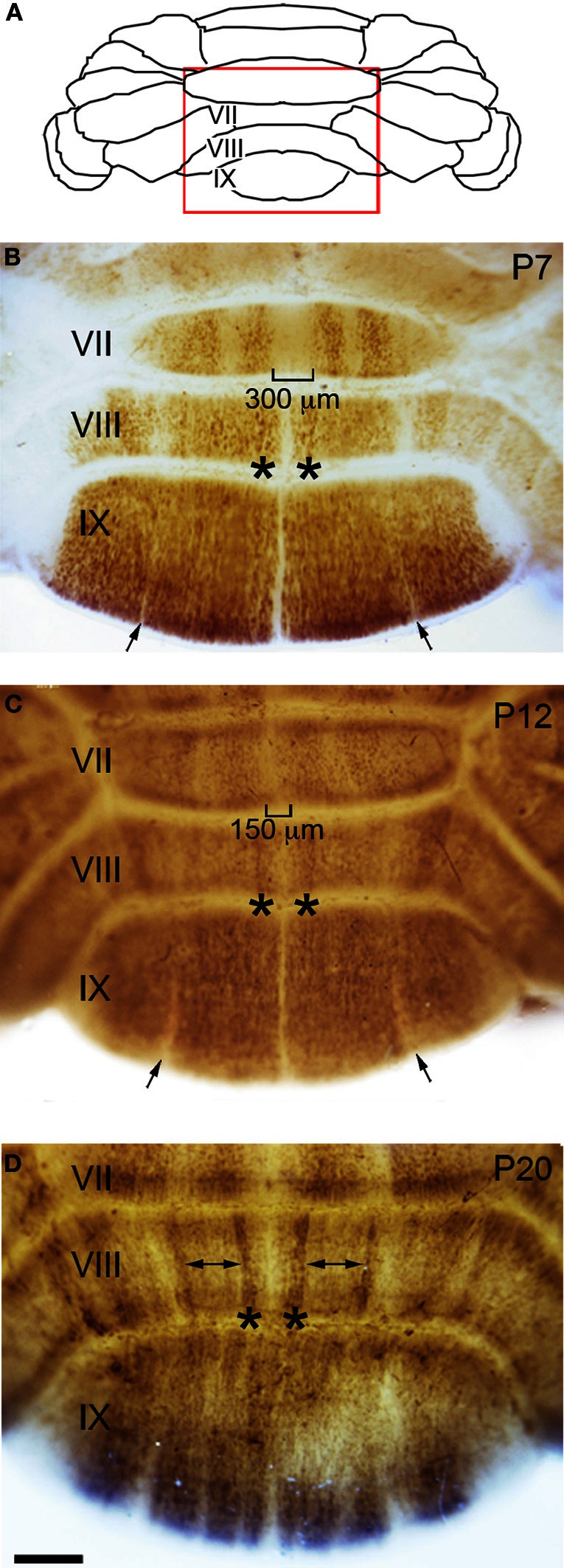
**Zone formation in the vermis revealed by NFH expression. (A)** Schematic indicating the region of the vermis that is shown in panels **(B–D)**. **(B)** At the end of the first postnatal week, NFH expression reveals clear zones in the posterior lobules. **(C)** By P12, the immunopositive zones in lobule VII have expanded laterally, and immunonegative zones decrease in width accordingly. This expansion is obvious at the midline (brackets). **(D)** By P20, strong expression at the lateral margins of each zone emerge (asterisks), while weaker expression persists within the center of each zone (double arrows). The negative zones in lobule IX have resolved by P20 (compare to narrow zones marked by arrows in panels **B** and **C**).

Sharp NFH zones resolve between P2 and P7. In addition, during postnatal week 1, specific subsets of lobules start to express NFH in unique patterns that respect the divisions of the four transverse domains (Figure 3; Ozol et al., 1999). In the anterior domain (lobules I–V; Figure 3), we observed two broad symmetrical zones of expression on either side of a negative midline. In the central domain (lobules VI–VII; Figure 3), the pattern of NFH is very complex as its expression demarcates two “sub-regions.” The first sub-region comprised lobules VIa–VIb, which contains symmetrical zones located immediately adjacent to the cerebellar midline, and on each side an additional broad zone, ~500 μm wide, that extends laterally to the paravermis (Figure 3). Lobule VII constitutes the second sub-region and contains a pair of ~150–200 μm wide zones on each side of the midline (Figure 3). The division of the central domain into sub-regions is reminiscent of that previously observed for NFH in the adult (Demilly et al., 2011) and Neurofilament-associated antigen during development (Marzban et al., 2008). The PZ has a simple pattern of NFH zones at P7; one very wide zone (~500 μm) that extends almost half the width of lobule VIII vermis, with an additional narrow zone ~150 μm wide located laterally (Figure 4B). Lobule IXa also has very wide zones that are interrupted by thin NFH-negative raphes (arrows Figure 4B, see below). In contrast, in the nodular domain lobule IXb contains a thin zone immediately adjacent to the midline, and additional 1–2 zones laterally (Figure 3). Although NFH is widely expressed in Purkinje cells of lobule X, weak stripes of NFH are consistently detected. Like the central domain, the nodular domain has sub-regions: lobule IXb has at least six zones (Figure 3) while lobule X has poorly resolved zones because most Purkinje cells express similar levels of NFH (Figure 3).

**Figure 5:**
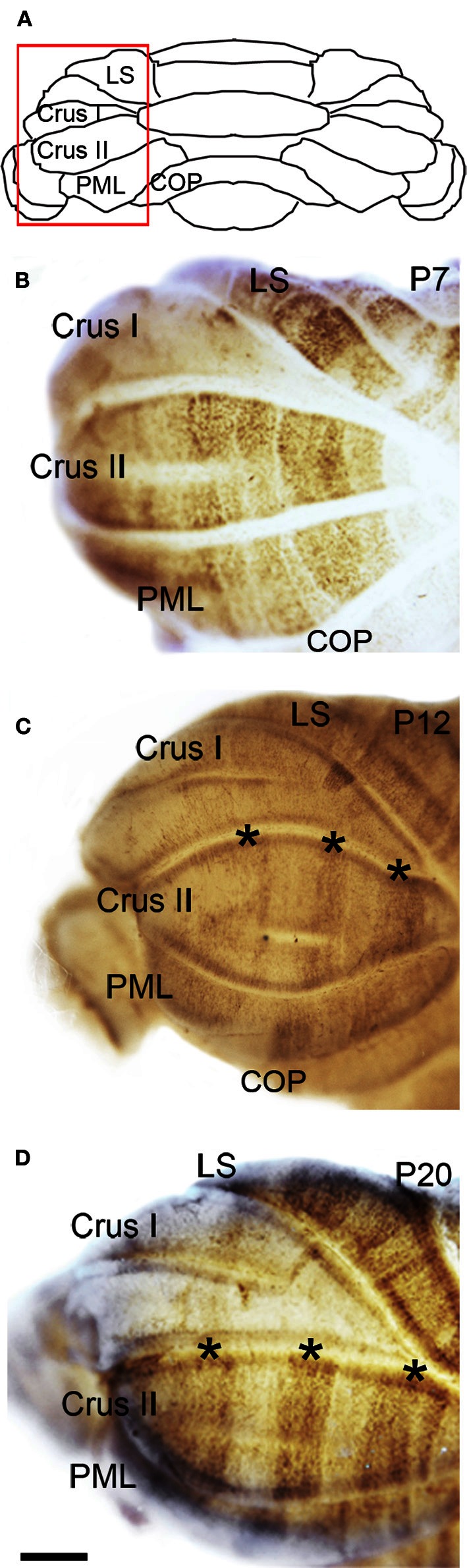
**NFH zone boundaries are sharpened in the developing hemispheres. (A)** Schematic indicating the region of the hemisphere that is shown in panels **(B–D)**. **(B)** NFH expression reveals a raphe-like pattern in Crus II at P7. **(C)** By P12, the NFH pattern consists of clear-cut zones (asterisks in **C** and **D**). **(D)** The overall pattern at P20 is identical to P12, although each zone is now sharply delineated. Abbreviation: LS, lobulus simplex; PML, paramedian lobule; COP, copula pyramidis. Scale bar = 500 μm.

The hemisphere lobules, LS, Crus I, Crus II, PML, and the COP reveal a complex NFH pattern that is comprised of a series of raphe-like gaps of weakly stained Purkinje cells (Lin and Cepko, 1998; Karam et al., 2000; Luckner et al., 2001; Furutama et al., 2010, Figure 5). In addition, the LS, Crus II, and PML express high levels of NFH whereas Crus I and the COP express low levels of NFH protein (Figure 5B). Together, these data reveal that zones of NFH expression are initiated soon after birth, and by P7 complex patterns have emerged in both the vermis and hemispheres.

#### Postnatal week 2

The overall pattern of NFH does not change between P7 and P12. However, in the hemispheres we observed a transformation from raphe-like gaps to zones (compare P7 and P12, Figures 5B,C). Several transformations also occurred in the vermis. The medial zone in the vermis of Lobule VII expands toward the midline, and as a result the ~300 μm NFH-negative zone observed at P7 is reduced to ~150 μm at the cerebellar midline at P12 (Figure 4C). The medial margin of the midline zone of lobule VIII exhibits an increase in NFH intensity between P7 and P12 (compare asterisks in Figures 4B,C). Similar to the hemispheres, the thin raphes of lobule IX give way to broader zones (arrows Figure 4C, and compare Figures 3F,I). These data indicate that map development during the second postnatal week involves the addition of new zones and the refinement of existing zones that are established during the first week after birth.

#### Postnatal week 3

There are two major changes that occur in the pattern of NFH between P12 and P20: (1) the intensity of NFH expression within zones increases and (2) zonal boundaries sharpen dramatically in both the vermis and the hemispheres. For example, at P20 the vermis of lobule IX is organized into clear zones, and the hemisphere zones extend over several lobules curving with the prominent contours of Crus II and the PML (Figures 3 and 5). Perhaps the most striking patterning change that occurs by P20 is within the midline zones in Lobule VIII. The center of each zone is comprised of Purkinje cells that express NFH at moderate levels whereas the medial and lateral edges of each zone are defined by Purkinje cells that express NFH at high levels (double-headed arrows in Figure 4). By P20, the zonal pattern of NFH is clear in all lobules (Figures 3–5; Demilly et al., 2011).

### NFH zones respect a fundamental molecular topography in the developing cerebellum

**Figure 6:**
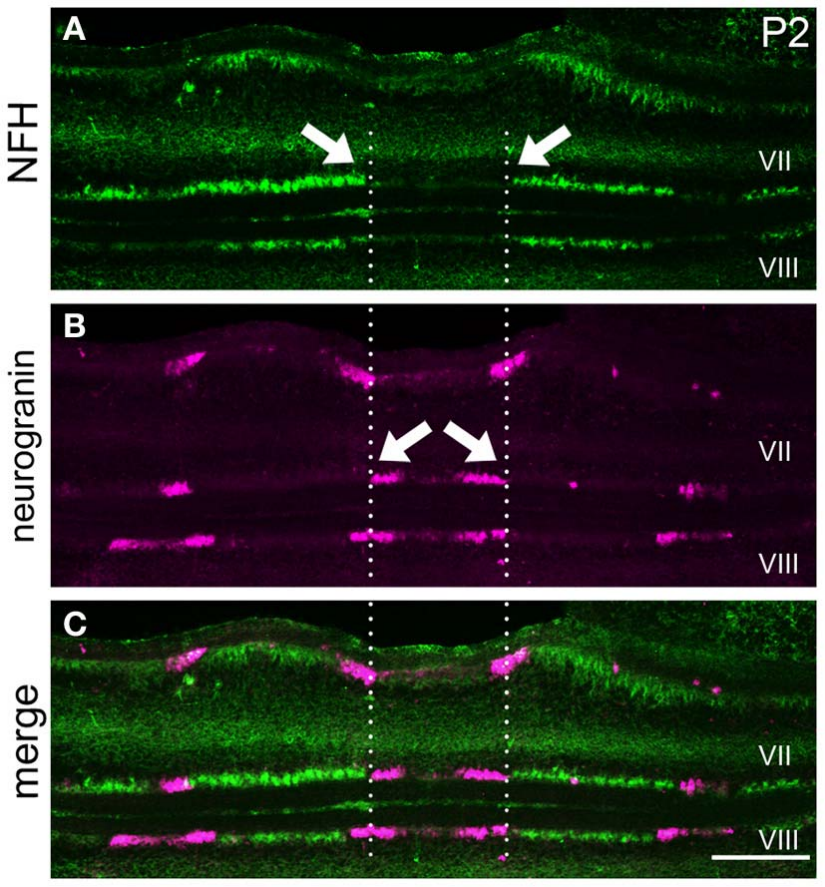
**NFH and neurogranin are expressed in complementary zones. (A)** NFH is expressed in broad zones at P2. **(B)** Neurogranin is expressed in a series of early postnatal Purkinje cell zones. **(C)** The pattern of NFH is complementary to the pattern of neurogranin at P2. The arrows point to zonal boundaries and the vertical dotted lines highlight complementary domains at the midline. Scale bar = 250 μm.

**Figure 7:**
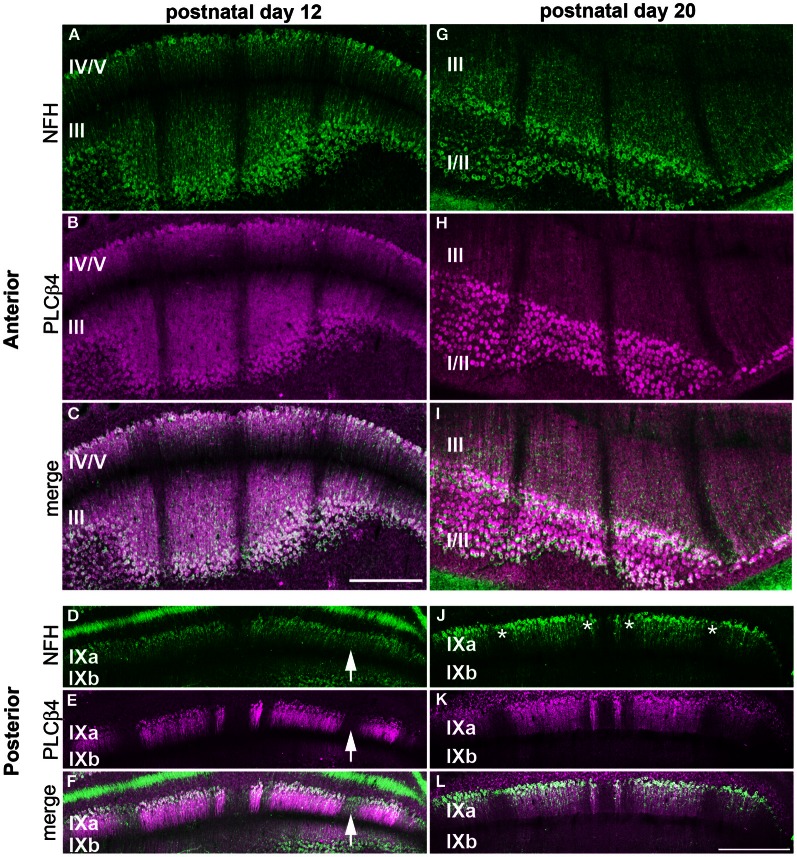
**NFH and PLCß4 have a complex zonal relationship.** NFH (green) and PLCß4 (magenta) have corresponding patterns in the anterior lobules at P12 **(A–C)** and P20 **(G–I)**. **(D–F)** In the posterior cerebellum, the widespread expression of NFH overlaps with PLCß4 positive and PLCß4 negative zones (arrows). **(J–L)** By P20, all NFH positive and negative zones (asterisks) overlap with PLCβ4 zones. Scale bar in **(C)** = 500 μm [applies to **(A,B,G–I)**] and the scale bar in **(L)** = 500 μm (applies to **D**–**F**, **J**,**K**).

Our finding that NFH is expressed in specific zones from ~P2 onwards suggests that its pattern might respect the fundamental molecular topography that was previously observed during early postnatal development (Larouche et al., 2006; Marzban et al., 2007). Indeed, we found that, at P2, NFH and neurogranin zones are largely complementary in the PZ with only minimal overlap observed between the two maps (Figure 6). In addition, we found that the relationship between NFH and PLCß4 changes over time. At all stages examined, we found extensive overlap in the anterior lobules (compare P12 to P20 in Figures 7A–C and G–I). In contrast, at P12 in the posterior lobules NFH is expressed in both the PLCß4-positive as well as the PLCß4-negative zones (arrows Figures 7D–F). Only after P15 did the expression patterns correspond in the posterior lobules (see P20 in Figures 7J–L). These data demonstrate that although NFH expression is spatially and temporally dynamic, the boundaries of its zones respect a topographic framework that is common to other known molecular markers.

## Discussion

The postnatal morphogenesis of cerebellar zones is poorly understood. In this study, we found that NFH, a member of the neurofilament cytoskeletal protein family, is expressed within Purkinje cell zones during early postnatal development. We revealed that during the first three weeks of life the maturation of NFH expression into adult zones mainly involves boundary refinement rather than gross reorganization of the overall pattern. Moreover, we show that during development, NFH zones respect the same medial-lateral boundaries as two known markers of postnatal zones, neurogranin and PLCβ4. Despite the protracted period of boundary refinement, the pattern of NFH expression falls into a well-understood framework of cerebellar sagittal zones.

### Establishment of the NFH pattern is spatially and temporally dynamic

We previously showed that, in the adult, NFH is expressed in a unique pattern of Purkinje cell zones (Demilly et al., 2011). In this study, we demonstrate that NFH is detected in Purkinje cells after P0, and that its pattern of expression is remarkably dynamic during development. We observed a nascent zonal configuration at P2/3 (Figure 2), and more sharply defined zones at P7 (Figures 3, 4, 5). By P12, we found that the zonal pattern of NFH in the hemispheres resolves into the bold pattern that is characteristic of the adult (Figures 3 and 4, Demilly et al., 2011). The adult pattern of NFH in the posterior and nodular zones is established late, and clearly demarcated zones were evident by ~P20 (Figure 3). This is not the first demonstration of a protein that exhibits such a dynamic pattern of expression during development. HSP25, for example, initiates in a pattern of Purkinje cell zones at birth; after ~P6 it enters into a period of global expression (Armstrong and Hawkes, 2000; Armstrong et al., 2001). After ~P10, HSP25 expression is suppressed selectively in a subset of Purkinje cells, which allows zones to emerge only in the central and nodular domains. The mature pattern is established after ~P15 (Armstrong and Hawkes, 2000; Armstrong et al., 2001). There are some key similarities between the spatiotemporal dynamics of NFH and HSP25 expression. Both of these proteins are expressed in Purkinje cells after birth, both are initiated in zones during the first postnatal week, and the mature zonal patterns that are characteristic of each protein are established after P15. However, NFH and HSP25 follow different mechanisms of zone maturation (refinement versus re-patterning, respectively), although the timing of when each transition takes place occurs at nearly identical times. Taken together, these data suggest that zonal patterning is influenced by two general regulatory mechanisms. One is a spatial mechanism that determines the precise organization and anterior-posterior location of specific zones and the other is a temporal mechanism that specifies when each zonal pattern is initiated and refined.

### The postnatal expression pattern of NFH respects a well-known purkinje cell topography

Neurogranin and PLCβ4 expression, fate mapping using an *L7/Pcp2-CreER* allele, and lineage tracing using a viral strategy that labels Purkinje cells on the day they are born all suggest that positional information contained within Purkinje cell clusters is transferred to the pattern of adult Purkinje cell zones (Larouche et al., 2006; Marzban et al., 2007; Sillitoe et al., 2009; Namba et al., 2011). Recently, an in depth expression analysis of FoxP2, PLCβ4, EphA4, Pcdh10, and an *Ip3r1* reporter transgene allowed embryonic clusters to be followed into perinatal parasagittal zones (Fujita et al., 2012). Despite these advances in our understanding of the embryonic origins of parasagittal Purkinje cell stripes, we do not fully understand whether different markers respect the same zonal boundaries during the critical stages of postnatal circuit development: from birth to P21. We show that NFH expression has a complex relationship to neurogranin and PLCβ4 (Figures 6 and 7). Importantly, the expression of all three markers together provides a means of visualizing the developmental zone map from birth through to full maturation of the functional topographic map. Remarkably, although NFH, neurogranin and PLCβ4 appear to respect the same spatial boundaries during development, the patterning of zones in each transverse domain follows a unique temporal progression. For example, whereas the anterior domain patterns of NFH and PLCβ4 fully correspond and are unchanged from birth to adulthood, the changes in NFH expression in the PZ result in temporally specific relationships with the overall pattern of PLCβ4 (Figure 7). Our finding that the temporal changes of NFH expression respect the boundaries of the transverse domains provides further evidence that each domain is a unique developmental compartment within the cerebellar cortex (Ozol et al., 1999).

### Postnatal molecular expression patterns define distinct stages of cerebellar development

The zonal organization of Purkinje cells is thought to guide the formation of patterned topographic circuits (Apps and Hawkes, 2009). During the first, second and third postnatal weeks zone formation progresses through multiple stages that ultimately produce the adult expression patterns at ~P15 (e.g., NFH, ZebrinII, HSP25). It is intriguing that each of these periods coincides with specific stages of circuit formation: mossy fiber afferent arrival during the first week (Ashwell and Zhang, 1992), afferent pruning and translocation of mossy fibers to granule cells during the second postnatal week (Hashimoto et al., 2009; Kano and Hashimoto, 2009), and the completion of climbing fiber pruning during the third week after birth (Hashimoto et al., 2001; Bosman et al., 2008). The direction of control is proposed to go from Purkinje cells to afferents. However, changes in Purkinje cell patterns have been observed as a result of a mutation that affects calcium signaling (Miyazaki et al., 2012). Afferent activity drives postsynaptic calcium signaling by activating several classes of ion channels and cell surface receptors. Given that NFH expression is sensitive to activity (Duffy and Livingstone, 2005; Duffy et al., 2007), it will be interesting to determine whether the temporal coding of NFH zones requires afferent dependent neural activity. Visual deprivation alters the expression profile of NFH in the cat lateral geniculate nucleus (Bickford et al., 1998), and also in the primary visual cortex of monkeys (Duffy and Livingstone, 2005) and humans (Duffy et al., 2007). We speculate that Purkinje cell zone refinement may provide a read-out of the developmental milestones that establish functional cerebellar circuits.

### Conflict of interest statement

The authors declare that the research was conducted in the absence of any commercial or financial relationships that could be construed as a potential conflict of interest.
